# Collagenase Management of Multicord Dupuytren’s Disease under Intravenous Sedation: A Prospective Cohort Study

**DOI:** 10.1097/GOX.0000000000002133

**Published:** 2019-02-20

**Authors:** Jeremy Wiseman, Kevin Tree, Pedro Guio-Aguilar, George Pratt, Danielle Nizzaro, Michael Leung, James Leong

**Affiliations:** From the * Department of Plastic Surgery, Monash Health, Dandenong Hospital, 135 David Street, Dandenong North, Victoria, Australia 3175; † Plastic Surgery Unit, Department of Surgery, Monash University, Wellington Rd., Clayton, Victoria, Australia 3800.

## Abstract

**Background:**

Surgery has been the standard of care in managing Dupuytren’s disease (DD). Recently collagenase of *Clostridium histolyticum* (CCH) has provided a less invasive alternative. The purpose of the current study is to present the early outcomes of a protocol for CCH involving treatment of multi-cord disease, and large patient cohorts.

**Methods:**

A cohort of 137 consecutive patients (Mean age 66 years, SD 9.85) with 225 joint contractures was treated with CCH at our institution between December of 2014 and January of 2017. A single standardized concentration of collagenase 2.31 mg/ml or 0.58 mg/dose was used for the treatment of up to 5 cords at a single session, and manipulation was 48 hours post-injection under intravenous sedation (IV). Patient complications, reduction in joint contracture, patient satisfaction and patient reported functional outcomes were assessed after one month.

**Results:**

137 patients received a total of 214 doses 0.58mg of CCH to treat 225 PIP and MCP joint contractures. The mean correction of joint contractures was 39.8 ± 2.2 and 27.9 ± 3.9 degrees for MCP and PIP joints respectively. 80% of patients, reported improved function and 89% of patients who were satisfied with the treatment.

**Conclusions:**

This study demonstrates a protocol for high throughput management of DD using collagenase and IV sedation for manipulation, logistically suited to the hospital setting. Efficacy was demonstrated treating patients with up to 5 cords, including those with bilateral disease. Future studies are needed to evaluate the durability of response in the medium and long term, and to evaluate cost benefits.

## INTRODUCTION

Traditionally, the management of Dupuytren’s disease (DD) has incorporated various surgical interventions ranging from percutaneous needle aponeurotomy to open dermatofasciectomy, necessitating skin grafting, occasional flap reconstruction, or open wounds left to heal by secondary intention, as described in the open palm technique by McCash.^1^ Standard surgical management can be challenging, and there are issues with recovery such as stiffness, swelling, prolonged wound healing, and intensive hand therapy.^2^ Surgery carries risk complications most notably neurovascular injury, a risk that increases with revision surgery.^2–4^ Multiple nonsurgical interventions have been trialed with limited success,^5^ but in 2010, the Food and Drug Administration (FDA) approved the use of a nonsurgical alternative for the management of DD as injectable *Clostridium histolyticum* collagenase enzyme (CCH) (XIAFLEX; Actelion Pharmaceuticals, Belrose, New South Wales, Australia). The current FDA-approved protocol for the use of CCH involves up to 3 injection cycles, using an average of 0.99 mg of enzyme without accounting for drug wastage, and was designed for the treatment of a single cord.^6,7^ Consequently, application to clinical practice in the public hospital setting has been challenged by questions of cost efficacy, which has yet to be convincingly demonstrated in the literature.^8–11^ Exploration into off-label treatment protocols for collagenase aimed at increasing the efficiency of collagenase treatment is underway, and the current study follows in this vein.^12–15^ Clinical and toxicological studies have suggested that higher total doses than those approved by the FDA are safe for use, and emerging reports of multiple injections over several years have demonstrated no systemic adverse reaction.^16–19^ The objective of the current study was to evaluate a protocol aimed at increasing the efficiency of managing patients with CCH to increase the suitability of this treatment option for the public healthcare system. To this end, we have employed the use of batch dosing, standardized dilution, multicord, injections, and intravenous sedation.

## PATIENTS AND METHODS

### Study

A single-institution prospective single-arm observational study was designed to evaluate the safety, efficacy, and applicability of a modified protocol for the use of CCH in the Australian public hospital setting. The study was approved by our institutional Human Research Ethics Committee, and CCH (XIAFLEX) has been approved for use in Australia by the Therapeutic Goods Administration. Between December 2014 and January 2017, a total of 137 patients (79.5% male) received injections of CCH according to the protocol described below (Table 1) for metacarpophalangeal (MCP) and proximal interphalangeal (PIP) joint contractures. Cords affecting the thumbs of study participants were treated, but data have been excluded.

**TABLE 1.**
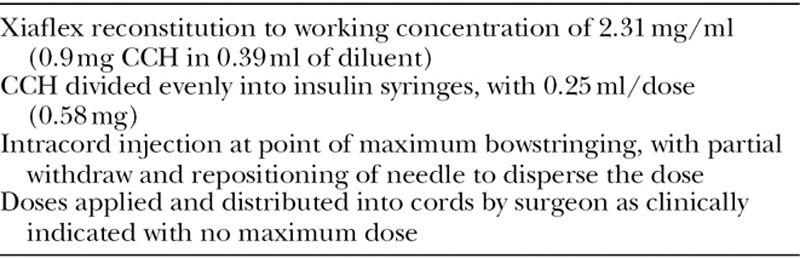
Modified Injection Protocol

### Patients

Patients on the waitlist for the management of DD were invited to participate in this study if they were over the age of 18 years and possessed a palpable cord causing joint contracture of >20 degrees of active extension at ≥1 MCP or PIP joints. Patients were excluded if they were pregnant or breastfeeding. Patients with multiple joints and rays affected, bilateral disease, or medical comorbidities (Table 2) were included in our study. All participants were provided with an information sheet approved by an institutional review committee, including details of the off-label nature of the study protocol, associated risks, and signed consent forms before treatment.

**TABLE 2.**
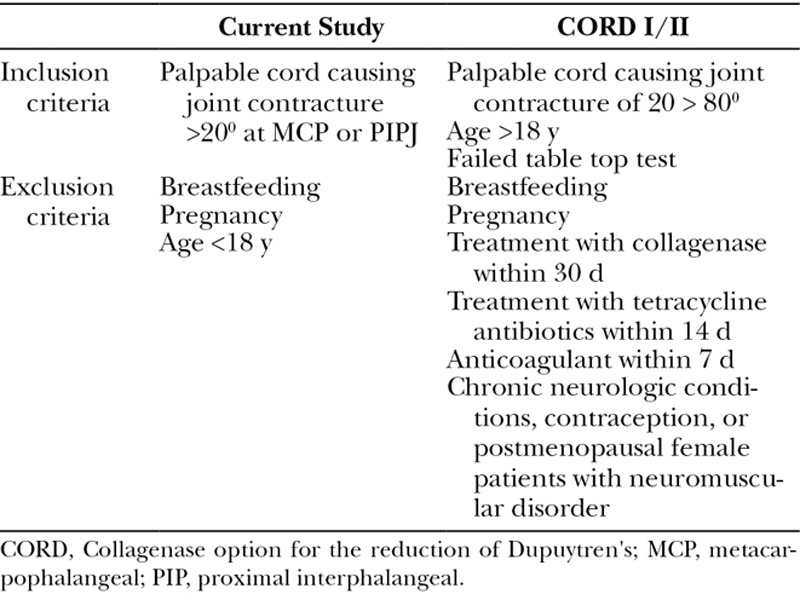
Modified Versus CORD I/II Selection Criteria

### Protocol

For the modified protocol, CCH was diluted to a single working concentration of 2.31 mg/ml or 0.39 ml of diluent for each bottle of CCH containing 0.9 mg of enzyme. The diluted enzyme was immediately divided using insulin syringes into aliquots (0.25 ml) or doses on the day of injection. Vials were shared to avoid wastage of residual drug which resulted in an additional dose for every 2 vials used.

The injection technique was modified from the previous study protocols by limiting injection to a single intracord injection, with partial withdrawal and repositioning of needle distributing the dose as evenly as possible within each cord (Table 1). Up to 5 separate doses of CCH (0.58 mg) were used per patient. In patients with multicord disease, the most appropriate cords were injected, as determined by the surgeon at the time of treatment. No local anesthetic was used at the time of enzyme injection.

In the present study, 5 cohorts of up to 35 patients received treatment in the outpatient clinic setting of an Australian public hospital, with a single session allocated to the treatment of each cohort. Forty-eight hours after injection, all patients returned to hospital for admission and manipulation under IV sedation in the operating room. Briefly, patients were admitted, cannulated for IV access, and then transferred to the operating theatre where they received propofol sedation without any local anesthetic. Manipulation of treated joints was carried out, and patients were moved to recovery for routine postanesthetic observation and subsequently discharged. The entire treatment group of 28–35 patients was completed within 2–4 hours during a morning theatre session. Injections and manipulation were carried out by one of the same surgeons who administered the collagenase injections. All patients were reviewed in outpatient clinic the following week. Any patients with large (>1 cm) skin tears received a 5-day course of oral antibiotics and simple dressings before discharge. All patients were provided with an information sheet containing prescribed postoperative hand exercises.

Clinical data were gathered preinjection, at the time of manipulation, at 1 week, and 1 month postmanipulation. Ongoing data collection includes 1-, 2-, and 5-year time points for long-term follow-up. In the present study, as a primary endpoint, the authors measured MCP and PIP joint goniometry, with a standard goniometer at maximum passive extension. Patient-reported outcomes were recorded using a disease-specific functional questionnaire (Unité Rhumatologique des Affections de la Main URAM) and a categorical patient satisfaction questionnaire. All immediate and delayed complications of the treatment were recorded, and an on-table evaluation of manipulation outcome was made by the surgeon.

## RESULTS

Between 2014 and 2017, 137 patients, 109 men and 28 women, were treated with CCH in the public hospital (Table 3). According to the described protocol (Table 1), a total of 214 doses (0.58 mg/dose of CCH) were used to treat 225 joint contractures in 191 rays (Table 3). The mean age of our study participants was 66 ± 9.85 years.

**TABLE 3.**
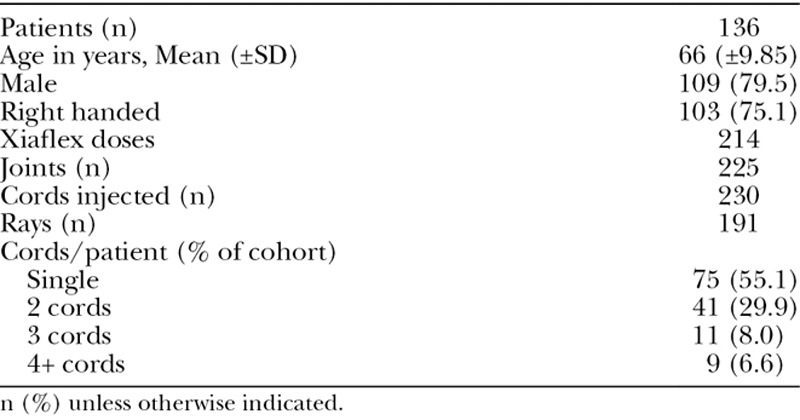
Baseline Population Characteristics

**Fig. 1.**
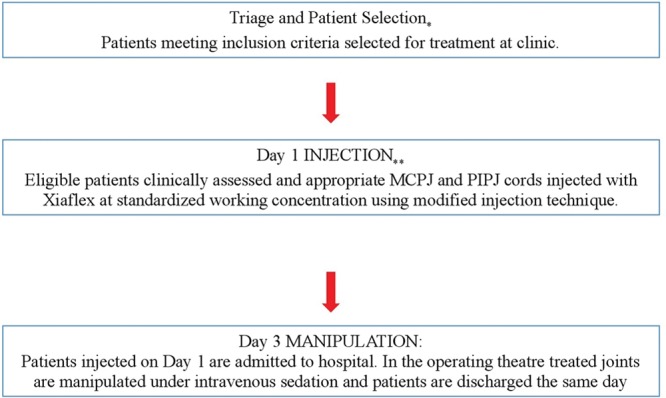
The modified protocol: *patients triaged and selected according to study inclusion criteria as potential candidates. **Day 1: patients attend outpatients’ clinic to undergo clinical assessment and selection of cords for injection as deemed appropriate by surgeon, and injection with premixed Xiaflex (CCH) at standardized dose and working concentration. MCPJ, metacarpophalangeal joint; PIPJ, proximal interphalangeal joint.

The described protocol utilized a high-throughput treatment cycle for the management of patients with CCH in the public hospital setting, which allowed for the management of up to 35 patients in a single morning session (Fig. 1; Table 2).

On table, at the time of manipulation, complete passive extension was achieved in 84% (188/225) of joints treated, partial response was seen in 13% (28/225), and no improvement was seen in 3% (8/225) of patients (Table 4). All patients experienced the previously described side effects of bruising, swelling, and some degree of pain postinjection, but all settled by the time of review at 1 week (data not shown). There was no major systemic adverse event. An immediate local adverse event of PIP joint dislocation occurred in a single patient (Table 3). This was managed conservatively with splinting, and the patient subsequently made a full recovery. Consistent with the previous studies, 225 joint manipulations resulted in 5.4% (n = 12) major (>1 cm) and 18.8% (n = 42) minor (<1 cm) skin tears (Table 4). All healed completely with dressings. Two patients experienced minor infection and were treated with a single course of oral antibiotics. The number of skin tears in patients treated for single cord disease was similar to those who received multiple injections for multiple cords (Table 4). At 1 month postinjection, 89% (76/85) of patients who completed the questionnaire reported being at least “Quite satisfied” with the treatment (data not shown). Using the previously validated URAM patient-reported functional questionnaire, 80% (73/91) of patients who responded demonstrated mean improvement of 25% (11.1/45) in their score (data not shown, Table 4).

**TABLE 4.**
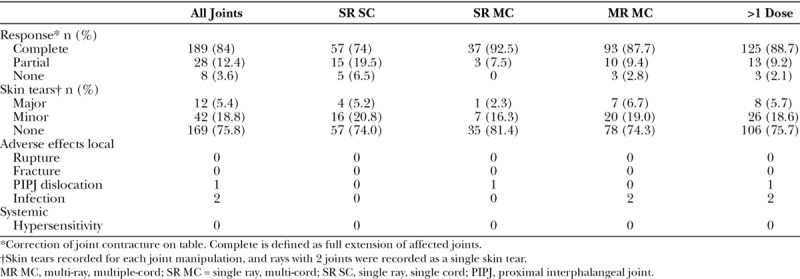
Immediate Response and Treatment-Related Complications

Correction of joint contracture in MCP and PIP joints, reported as the mean across the entire study population, is presented allowing comparison of the current study with the registration trials CORD I and II. Goniometry at 30 days after manipulation revealed an efficacy equivalent to results achieved by CORD I and CORD II studies, using 41% less drug (Table 5).^6,7^ There was on average 39.8 ± 2.2 degrees of correction in MCP joints and 27.9 ± 3.9 degrees of correction per 0.58 mg dose for PIP joints.

**TABLE 5.**
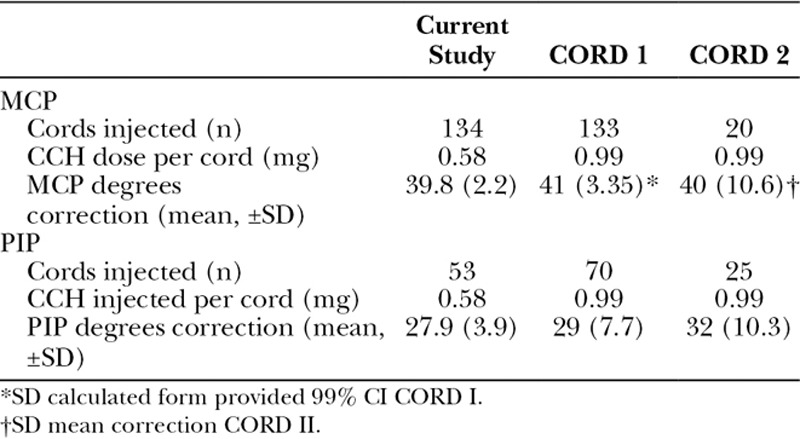
Efficacy of CCH Injections for MCP and PIP Joints at 30 Days After Manipulation

## DISCUSSION

The efficacy of collagenase as a nonsurgical intervention was convincingly demonstrated through randomized double-blinded, placebo-controlled trials in 2009 and 2010, and subsequent to regulatory approval, there have been a steadily increasing number of reports on the use of CCH. Recently, published studies have moved toward the evaluation of various off-label modifications of the FDA-approved protocol for the use of CCH.^6,7,12–15,20^

FDA approval for the use of CCH describes the treatment of a single cord with a single dose, prescribing repeat injections after 30 and 60 days if success was not achieved with the first injection. Recent trials on the safety of treating single and multiple cords in both MCP and PIP joints have been published; however, to date, these studies are limited to the treatment of 2 cords or the use of a single vial per hand.^12–15^ The present study extends this body of evidence to the treatment up to 5 cords in both unilateral and bilateral diseases during a single session. The current study demonstrates equivalent clinical efficacy, as gauged by goniometry of joint contractures mean 39.8 ± 2.2 degrees (MCP joints) and 27.9 ± 3 degrees (PIP joints), rivaling results achieved in the CORD I and II studies. Notably, the results demonstrated in the current study were achieved with a greatly reduced enzyme dosage per cord, 0.58 mg versus 0.99 mg used in the CORD studies (Tables 4, 5).^6,7^ Modifying the injection technique and to use a single intracord injection similar to that published by Verheyden^12^ has likely contributed to increased efficiency, indeed Verheyden^12^, Grandizio et al.^13^ among others demonstrated similar levels of success correcting joint contractures with lower average enzyme doses.

To measure functional outcome for the patient, the previously validated URAM functional questionnaire was used.^21^ In addition to correction of joint contractures, scores for patient-reported functional outcomes improved.

Forgoing local anesthesia and substituting IV sedation during manipulation of injected cords allowed for an efficient, high-throughput treatment model that, when optimized to the healthcare provider, is well suited to the public hospital setting. The use of intravenous sedation and intraoperative manipulation of cords remains unreported, and evaluation of patient satisfaction was high, lending support to the use intravenous sedation without local anesthesia.

The relative cost of collagenase versus surgical and percutaneous management of DD has been scrutinized in the literature. Analyses from the United States, Canada, Spain, and Sweden have reached a similar conclusion that current US market prices for CCH do not allow accepted threshold for cost efficacy to be reached.^8,9,11^ Other reports in the literature, however, have demonstrated CCH to be economical. Atroshi et al.^10^ published a report in 2014 evaluating direct costs for CCH versus fasciectomy and found CCH to be cheaper with similar efficacy at 6 weeks. A cost–benefit analysis of the current protocol has not been applied here; however, the ability to treat up to 30 patients per session using less enzyme per cord than FDA-approved protocol has generated positive revenue for our healthcare network (data not shown) and furthermore a reduction of overburdened waitlists, which suggests a strong potential for cost efficacy within the Australian public health framework.

This study was limited by low response rates to the questionnaires and lack of statistical powering to objectively demonstrate safety; however, it demonstrates a protocol for the management of multiple patients with DD using intravenous sedation, with a rate of success equivalent to published data.^6,7,12^ Further, this study has shown the treatment of up to 5 cords and bilateral disease in a single treatment session without major adverse events. Future studies will be required to evaluate the durability of response in the medium and long term, the relative efficacy of single versus multiple joint treatments, and formally, the cost efficacy.
